# English Hotels and Their Critics

**Published:** 1889-07-20

**Authors:** 


### EDITOR'S LETTER-BOX.

[Our Correspondents are reminded that prolixity is a great bar to publication, and that brevity of style and conciseness of statement greatly facilitate early insertion.]

### ENGLISH HOTELS AND THEIR CRITICS.

Sir,—Through the medium of your widely-read publication, extracts from which are now frequently quoted by our leading dailies, I desire to solicit the attention of the medical profession to the dietariet associated with the hotels of our leading watering places; and in this connection I would venture to point out the manifest injustice so often unwittingly inflicted by many eminent medical advisers in recommending their patients to *quasi* popular health resorts without possessing any personal knowledge of the direful and innumerable petty inconveniences to which the unfortunate patient is subjected in our modern English hospice.

If careful nursing be half the cure, surely palatable, nutritious food, judiciously cooked and tastefully served, is the prime essential; and where, ye *savants*, within the borders of this island are all three indispensables combined?

Take the case of a delicately-nurtured young lady, accustomed in her own home to all that wealth and careful selection can supply. When overtaken by illness she, at the mandate of her medical adviser, is ordered change of air to one of our inland or seaside watering places; try to picture the contrast.

The long, uncomfortable railway journey is trying enough to the most robust, but rendered still more miserable by the absence, on arrival at destination, of every vestige of those little considerations which help to make life endurable.

The parched palate longs and hopes for a cup of nice refreshing tea. Alas, vain hope! The management contract for tea at 1s. 2d. per lb., that is, inclusive of 6d. duty, leaving 8d. per lb. to pay freight, profit, and brokerage for a compound at which a coolie would blush were he to offer it as tea.

The slice of thin dry toast is either a flabby, sickly-looking leaf of singed dough, or a flinty slab of charcoal suitable for treads of stairs where there is much traffic. The bread throughout hotels is most carefully elaborated to produce chronic dyspepsia or atrophy, and the only thing it *sustains* is the medical profession.

But, lo! there is the *table d'hote* so marvellously designed to promote the health and happiness of the invalid!

The soup, oxtail; which tails bluebottles had for days held high festival on, till Mr. Butcher offers them to large hotels and boarding houses at a *great* reduction. The fish, noble turbot! only brill to-day, but no doubt it will be fresh, probably just caught; we are close to the coast. How innocent! The management contract with a man residing at Lowestoft, Yarmouth, etc., for a basketful, whatever the catch, at a price, prime and offal; the inferior comes in useful as *fillets de place* for commercials and servants. The rissoles very hot and piquant, artless little mysteries, like some of the fair ones who partake; but did you see the elements before they were transformed? No? Well, I did. Then approaches the grand *pièce de résistance*, the noble sirloin; he, indeed, must be an epicure who cannot satisfy his cravings on such a succulent *morceau*. Only mirage, my friend, which decoys the jaded wanderer. It is now "the old beef roast in England." That Butcher took shares in the big hotel hoping for a dividend. And so through each course *ad nauseam*.

Finally, a cup of coffee is suggested; no sooner ordered than, presto, it appears smoking hot. Oh, vile medicament! In no apothecary's most unsavoury "compounding department" could so nauseous a concoction be infused! Ye Mincing-lane brokers, do ye wonder that coffee sales decline while so base a substitute is allowed to vilify the name of delightful exhilarating Mocha? The liquors are similarly supplied by a syndicate, who "run" half-a-dozen hotels to dispose of their "rare old vintages" at a profit, and such a profit.

My contention is that modern hotel catering is a gigantic fraud, and that the *habitues* of such establishments, "replete with every comfort," are an ill-nourished, under-fed class, cheated of their daily bread so dearly paid for, developing a race of peevish, irritable dyspeptics; utterly unfitted for fighting the battle of life. It is not the long march which kills, but where the balance fails to be honestly maintained. The commissariat furnishes a larger death roll than the hand of the enemy. I therefore appeal to the members of that noble profession to use all their powerful influence in correcting, reforming, and refining the gross defects of our misnamed health resorts, and devote less of their professional acumen to the preparation of elaborate tables proving equability of temperature and salubrity of clime.

A bountiful Providence supplies these blessings with unstinted hand, but that curse of modern society, "the speculator," never rests till he perverts every natural advantage to a source of greedy gain.—Yours, etc.

